# Self-reported free-living physical activity and executive control in young adults

**DOI:** 10.1371/journal.pone.0209616

**Published:** 2018-12-26

**Authors:** Simon Ho, G. Kyle Gooderham, Todd C. Handy

**Affiliations:** Department of Psychology, University of British Columbia, Vancouver, British Columbia, Canada; University of Maiduguri College of Medical Sciences, NIGERIA

## Abstract

To what extent do our free-living physical activity (PA) levels impact our cognition? For example, if we engage in more intense PA from one week to the next, does this have a corresponding influence on cognitive performance? Across three studies, young adults completed a validated self-report questionnaire (the International Physical Activity Questionnaire, or IPAQ) assessing their involvement in PA at low, moderate, and vigorous intensities over the past week, as well as computer-based measures of executive control and attentional function. In Experiment 1 we found no significant effect of PA intensity on any of our measures of executive control. In a pair of follow-up control studies we examined whether these null findings could be attributed to testing fatigue and task complexity (Experiment 2), or low cognitive demands of the task (Experiment 3). Despite simplifying the task, reducing testing time, and increasing the cognitive load of the task, we still found no significant impact of weekly PA intensity on our measures of executive control. Taken together, our results show that self-reported PA over the past week, at any intensity level, does not appear to have a substantive impact on executive control.

## Introduction

In general, research studying the relationship between physical activity (PA) and cognitive function in healthy young adults falls into two broad categories: the study of near-term or single/acute effects of PA, and the effects of more long-term or chronic/intervention-style PA. While these categories capture the timing of PA relative to cognitive testing, the dichotomy begins to break down when considering more intermediate timescales that are longer than single bouts but that do not necessarily qualify as chronic. This is an important issue because the impact of PA on cognition has been shown to be quite varied depending on when the PA occurred [1]. For example, acute PA that occurred immediately prior to cognitive assessment has been shown to improve cognitive function [2–4], but when cognition is assessed *during* an acute bout of PA, the impacts appear to be negative [5–7]. At longer timescales, while increased voluntary chronic PA over the past 3 and 10 years has a positive impact on cognitive function [8,9], findings from shorter PA interventions in young adults (3 weeks to 2 months) have produced more equivocal results [10–12]. While mixed results can be found within each temporal category, these findings highlight the importance of examining PA timescales separately as they may have different effects on cognitive function.

From a public health and physical activity promotion perspective, PA over the past 7 days may be a useful timescale to study. While studies have shown a cognitive benefit of short-term (acute) PA [2–4], it is not always possible to exercise immediately prior to a cognitive task. Similarly, promoting the cognitive benefits of long-term PA may not be an effective way to increase overall activity levels either, as it can be difficult to remain disciplined enough to exercise consistently for months prior to a cognitive task. 7-day PA is a middle ground between chronic and acute timescales, where individuals have control over their PA levels and general lifestyle choices, and may still be motivated enough to exercise consistently throughout the week. However, recent meta-analyses show that the majority of young adult studies examining the relationship between PA and cognition have looked at acute timescales, with few assessing the impact of long-term PA, and even fewer looking at 7-day PA [13,14]. In the present study we will be focusing specifically on free-living (voluntary and self-initiated) activities over the past 7-days, which we will refer to simply as free-living PA.

The impact of free-living PA on cognition has been studied objectively using accelerometers, showing improved attention capacity to be associated with longer time spent in moderate-intensity PA [15], however, free-living PA can also be assessed via subjective self-report. Questionnaires have benefits over objective methods as they can capture activities that cannot typically be measured using accelerometry, such as cycling and swimming. A number of self-report studies have shown that increased PA time over the past week is associated with improved executive control [16,17] and response monitoring [18]. It is important to note, however, that many studies using self-report free-living measures only assess the frequency of activity, or amount of time spent being active [16–22]. While PA duration is important, it is unlikely to fully capture the intricacies of recent activity. For example, 30 minutes of light walking is likely to have a different impact on cognition than 30 minutes of high-intensity interval training due to differences in PA intensity and energy expenditure. Research on acute and concurrent PA supports this idea, for example, cognitive performance has been shown to change in an inverted-U fashion as a function of PA intensity, with attentional resource allocation increased following medium-intensity PA, and decreased after high-intensity PA [23,24]. Similar findings have been observed during concurrent PA, with high-intensity PA resulting in longer reaction times and higher error rates on a variety of executive control tasks [25–28]. That PA intensities have differential effects on cognition underscores the importance of considering both duration and intensity when examining free-living PA. Furthermore, while free-living PA studies in young adults have examined a variety of cognitive domains, such as implicit learning [29] and memory [29,30], the majority of work has focused on executive control. Behavioral and neuroelectric indices of improved executive control, defined as the ability to purposefully inhibit automatic responses [31], have been positively correlated with higher levels of weekly PA [17,18,20,29]. However, as most free-living PA studies generally do not examine the impact of PA intensity, it remains unclear whether these benefits remain once the intensity of PA has been accounted for.

The goal of the present study is to examine the relationship between free-living PA over the past 7 days and executive control in young adults, while taking into consideration the energy requirements of the various activities performed throughout the week. Research on free-living effects and PA intensity lead to the prediction that increased moderate-intensity PA should have a positive association with performance, with little-to-no effect of low or vigorous intensity PA. Tasks requiring executive control are an ideal starting point for examining free-living effects as they have been associated with PA levels at other timescales [32–34], and PA has been found to benefit executive function more than other cognitive domains [35]. To that end, free-living PA was assessed across three experiments using self-report questionnaires, and PA intensities were then tested for their association with performance on executive control tasks.

## Experiment 1

We began by assessing the relationship between free-living PA and cognition using the Attention Network Test, which is designed to examine three facets of attention: alerting, orienting, and executive control. The goal was to understand which form of attention was most associated with weekly PA at different intensities.

### Ethics statement

Ethical approval was received through the University of British Columbia’s Behavioural Research Ethics Board, and written informed consent was obtained from each participant prior to the start of the study.

### Participants

We conducted a two-tail power analysis, using a small effect size estimate of 0.20, to determine minimum sample size for the study. Only a handful of studies have examined the relationship between free-living PA and behavioral measures of executive control in young adults, and they estimate the effect size to be in the range of 0.34–0.38 [20,29,36]. Due to the paucity of research on this topic, we also turned to a couple of key meta-analyses to determine our effect size target for power analysis. Those meta-analyses show that the relationship between acute PA and cognitive performance (e.g. executive control, working memory) has a mean effect size of 0.20 in young adults [1], but can be as high as 1.24 for cross-sectional PA studies depending on the cognitive test used [37]. Therefore, our choice of 0.20 should be considered a conservative lower bound estimate of the population effect size, chosen to minimize the likelihood of being underpowered. The analysis indicated that a minimum of 193 participants would be needed to achieve 80% power. A total of 267 participants were recruited from undergraduate psychology courses at the University of British Columbia and received course credit for their time. Participants were eligible to take part in the study if they were young adults (under 45 years of age) and physically able to take part in PA. Three participants were excluded due to computer problems that prevented data collection, and two participants were excluded for vision-related issues (e.g. recent surgery). Our final sample consisted of 262 participants (mean age = 20.44, *SD* = 2.65, 138 male).

### Apparatus

All tasks and questionnaires were displayed using a 19” LCD monitor with a resolution of 1280x1024. Data collection for the computer tasks was conducted using the open-source Cognitive Battery 3.2 software package [38], which utilizes Python 3.6.4 and Pygame 1.9.3 for stimulus display. The primary operating system was Windows 7. The details of the individual tasks are described for each experiment separately.

### International physical activity questionnaire

At the start of the session, participants completed the self-administered long-form of the International Physical Activity Questionnaire (IPAQ; http://sites.google.com/site/theipaq), which measured self-reported physical activity (PA) over the past week. The IPAQ has high reliability and validity when monitoring physical activity across diverse populations [39–41], and has been used to study a wide range of cognitive outcomes such as academic achievement [42,43], response monitoring [18], spatial priming [16], task switching [17], and functional and structural brain connectivity [21,44]. The long-form was chosen because the short-form of the IPAQ shows low correlation with objective measures and typically overestimates activity levels [45].

The IPAQ long-form assesses physical activity undertaken in the past week across several domains, including leisure time PA, domestic activities, work-related PA, and transportation-related PA. Activities of different intensities are reported for each domain. Weekly duration estimates are calculated by multiplying time spent in a typical day by number of days spent in the past week performing that activity. Weekly durations are then multiplied by MET values for the different activities [46] to calculate MET-minutes per week for each intensity level. METs are multiples of resting metabolic rate, and multiplying activity duration by a MET value effectively weights the activity by the energy required to perform it. After data aggregation, the IPAQ reports MET-minutes per week for three intensities: low, moderate, and vigorous, which are estimates of energy expended over the past week performing activities at those intensity levels. Additionally, the IPAQ provides a total PA measure, which captures MET-minutes per week regardless of intensity. For a detailed explanation of the scoring process, see the official IPAQ scoring protocol [47]. Some researchers have used an alternate scoring method for the IPAQ that categorizes individuals as sedentary or active based on recommended PA levels by the American College of Sports Medicine (ACSM). Active participants were those that had ≥ 5 days/week of moderate-intensity PA or ≥ 3 days/week of vigorous-intensity PA, while sedentary participants had ≤ 2 days/week of moderate- or vigorous-intensity PA [17,18,21,48]. Our analyses include this alternate scoring method for comparison purposes. Participant PA information can be found in Table 1.

**Table 1:** Participant characteristics for all three experiments.

	Experiment 1	Experiment 2	Experiment 3
**Sample size**	262	218	206
**Age Mean (SD)**	20.44 (2.65)	20.11 (1.87)	20.33 (2.71)
**Sex (M/F)**	138 / 124	48 / 170	51 / 155
**MET-mins/week (low)**	1282.78	2037.83	2076.12
**MET-mins/week (moderate)**	1072.53	1104.16	1243.81
**MET-mins/week (vigorous)**	1373.39	1359.24	1623.80
**MET-mins/week (total)**	3728.70	4501.22	4943.73

### Attention Network Test (ANT)

After the IPAQ, participants completed the Attention Network Test [49], which is designed to measure three aspects of attention: the alerting task measures the ability to maintain a vigilant and alert state during continuous performance; the orienting task measures the ability to select information at different spatial locations; and the executive control task measures conflict resolution and the ability to inhibit task-irrelevant information [50,51]. Each trial began with a fixation cross in the center of the screen, displayed for 400-1600ms. Next, a cue (an asterisk) was displayed for 100ms. Four cue types were utilized to capture different aspects of the attentional function: 1) no cue, where the fixation cross remained unchanged and thus the participant was not warned about stimulus onset; 2) central cue, where the fixation cross was replaced by the asterisk; 3) spatial cue, where an asterisk appeared at the location of the upcoming stimuli (either above or below fixation); and 4) double cue, where asterisks appeared both above and below fixation, informing the participant of upcoming stimuli but providing no information about the location. Flanker arrows (five horizontal lines with arrowheads) were shown 400ms after the cue and remained on screen until the participant made a response or 1700ms had elapsed. Participants were simply asked to use the left and right arrows keys on the keyboard to indicate the pointing direction of the central arrow. Arrow directions could either be leftward-congruent (all arrows pointing to the left), leftward-incongruent (center arrow pointing left, flanking arrows pointing right), rightward-congruent (all arrows pointing to the right), or rightward-incongruent (center arrow pointing right, flanking arrows pointing left). A neutral condition was also shown, where the central arrow was flanked by plain horizontal lines (no arrowheads). All cue and arrow conditions were equiprobable and presentation order was randomized.

Participants began with a practice block of 24 trials, and the main task consisted of three blocks, each with 96 trials. Alerting performance was calculated as the difference between the double cue and no cue conditions, orienting performance was the difference between spatial and center cue conditions, and finally, the executive control performance was the difference between congruent and incongruent arrow conditions. These difference scores were normalized by dividing the difference by the faster of the two conditions, for example, the executive control score was divided by the individual’s mean congruent reaction time. This process places the reaction time in the metric of, in this executive control example, the congruent condition and makes the value more interpretable. The normalized conflict score is interpreted as the magnitude to which the person’s incongruent responses were slower relative to their baseline congruent performance. More details of this task can be found in the original ANT development paper [49]. The full dataset can be found in S1 Dataset.

### Data analysis

We hypothesized that moderate-intensity PA in the past week would be related to cognitive function. Specifically, higher levels of moderate-intensity PA would be associated with improved performance on the executive control task. Therefore, multiple regression was used to determine the relationship between self-reported PA at each intensity levels (low, moderate, vigorous) and performance on the alerting, orienting, and executive control tasks. Participant age was used as a covariate as it has been shown to impact cognitive performance [52–54]. Specifically, the model (Model 1) predicted alerting/orienting/executive control performance from age, low-intensity MET-mins/week, moderate-intensity MET-mins/week, and vigorous-intensity MET-mins/week.

Previous studies have looked at the effect of chronic PA on ANT performance [8], however, their PA measures did not include intensity categories. The IPAQ also provides a total PA measure that is collapsed across intensity, so we included a second model (Model 2) predicting ANT performance from age and total MET-mins/week. This is to maximize comparability between studies to ensure observed effects are due to the timespan of the PA measure (chronic vs. free-living), rather than being caused by looking at different intensities of PA. Finally, some studies have used an alternate coding method for the IPAQ [16,18] that classifies individuals as sedentary or active based on ACSM recommendations (described in the previous section). For each of the ANT outcome measures, we included a third model (Model 3) predicting performance from age and ACSM category to ensure the IPAQ scoring method does not change observed relationships. Regression assumptions were checked by visual inspection of quantile-quantile and normality plots, and no violations were indicated. Furthermore, due to potential issues with collinear predictors, variance inflation factor (VIF) was calculated for all predictors in each of the models. All VIF values were within an acceptable range (< 1.5).

### Results

#### Alerting task

We tested the relationship between alerting performance and PA intensity using Model 1. None of the predictors were significantly related to alerting performance. Age: *β* = 0.04, 95% CI [-0.08, 0.17], *t*(243) = 0.65, *p* = .52, low-intensity: *β* = 0.02, 95% CI [-0.11, 0.14], *t*(243) = 0.24, *p* = .81, moderate-intensity: *β* = 0.10, 95% CI [-0.03, 0.24], *t*(243) = 1.51, *p* = .13, vigorous-intensity: *β* = -0.05, 95% CI [-0.18, 0.09], *t*(243) = -0.68, *p* = .50. Using Model 2, we tested the relationship between total PA and alerting. However, none of the predictors were significantly related to alerting performance. Age: *β* = 0.04, 95% CI [-0.08, 0.17], *t*(245) = 0.67, *p* = .51, total PA: *β* = 0.04, 95% CI [-0.08, 0.17], *t*(245) = 0.68, *p* = .50. Finally, we tested the relationship between ACSM category and alerting using Model 3. Again, none of the predictors were significantly related to alerting performance. Age: *β* = 0.05, 95% CI [-0.08, 0.17], *t*(245) = 0.75, *p* = .46, ACSM: *β* = -0.23, 95% CI [-0.50, 0.05], *t*(245) = -1.61, *p* = .11.

#### Orienting task

We tested the relationship between orienting performance and PA intensity using Model 1. None of the predictors were significantly related to orienting performance. Age: *β* = 0.02, 95% CI [-0.11, 0.14], *t*(243) = 0.24, *p* = .81, low-intensity: *β* = 0.04, 95% CI [-0.09, 0.17], *t*(243) = 0.62, *p* = .54, moderate-intensity: *β* = 0.06, 95% CI [-0.08, 0.19], *t*(243) = 0.85, *p* = .40, vigorous-intensity: *β* = 0.03, 95% CI [-0.10, 0.16], *t*(243) = 0.44, *p* = .66. Using Model 2, we tested the relationship between total PA and orienting. However, none of the predictors were significantly related to orienting performance. Age: *β* = 0.02, 95% CI [-0.11, 0.14], *t*(245) = 0.25, *p* = .80, total PA: *β* = 0.08, 95% CI [-0.04, 0.21], *t*(245) = 1.30, *p* = .20. Finally, we tested the relationship between ACSM category and orienting using Model 3. Again, none of the predictors were significantly related to orienting performance. Age: *β* = 0.01, 95% CI [-0.11, 0.14], *t*(245) = 0.22, *p* = .83, ACSM: *β* = 0.13, 95% CI [-0.15, 0.40], *t*(245) = 0.92, *p* = .36.

#### Executive control task

We tested the relationship between executive control performance and PA intensity using Model 1. None of the predictors were significantly related to executive control. Age: *β* = 0.03, 95% CI [-0.10, 0.15], *t*(243) = 0.41, *p* = .68, low-intensity: *β* = 0.06, 95% CI [-0.07, 0.19], *t*(243) = 0.89, *p* = .37, moderate-intensity: *β* = -0.07, 95% CI [-0.20, 0.07], *t*(243) = -0.98, *p* = .33, vigorous-intensity: *β* = -0.003, 95% CI [-0.14, 0.13], *t*(243) = -0.05, *p* = .96. Using Model 2, we tested the relationship between total PA and executive control. However, none of the predictors were significantly related to performance. Age: *β* = 0.03, 95% CI [-0.09, 0.15], *t*(245) = 0.47, *p* = .64, total PA: *β* = -0.02, 95% CI [-0.14, 0.11], *t*(245) = -0.25, *p* = .81. Finally, we tested the relationship between ACSM category and executive control using Model 3. Again, none of the predictors were significantly related to executive control. Age: *β* = 0.03, 95% CI [-0.09, 0.16], *t*(245) = 0.54, *p* = .59, ACSM: *β* = -0.21, 95% CI [-0.49, 0.07], *t*(245) = -1.50, *p* = .14.

### Discussion

Overall, our results fail to support the hypothesis that moderate-intensity free-living PA is related to executive control. Previous studies have shown a relationship between chronic PA and executive control [8], however, our models utilizing the IPAQ’s total PA measure (Model 2) failed to show a relationship with any of the outcome measures. This difference in results suggests that the timescale of PA assessment may be important for PA and cognition effects. Furthermore, other studies have utilized different methods of coding the IPAQ based on ACSM recommendations [16,18]. Although those researchers focused on different cognitive outcomes, our results using Model 3 suggest that IPAQ coding method does not play a major role in identifying relationships between free-living PA and executive control.

The ANT is complex owing to its multiple cue and congruency conditions, and this complexity necessitates the use of many trials to establish stable condition means, resulting in long testing sessions (lasting almost 2 hours) and possibly fatigue effects. We examined the linear trend in participant reaction times across their 288 ANT trials by regressing reaction time on trial number, and calculating a regression coefficient for each participant, which represents the degree to which the individual’s responses were slowing down over time. The mean coefficient value suggested that, on average, participant responses were getting slower by 0.09ms for each successive trial. When these coefficient values were submitted to a one sample t-test (tested against 0), we found a significant degree of response slowing over time, *t*(261) = 3.82, *p* < .001. While not conclusive, this suggests the task length may be causing fatigue and a general slowing of performance, and possibly masking the impact of PA on executive control.

## Experiment 2

The results from Experiment 1 showed no relationship between free-living PA and executive control, however, there may be a fatigue effect due to the complexity and length of the ANT task. Previous studies have shown that chronic PA impacts only the executive control task in young adults [8], and similar results have been shown for acute bouts of PA using the Eriksen Flanker task [32]. The Eriksen Flanker task is one of the most commonly used measures of executive control in both the young adult PA literature [4,7,33,55,56], and PA intervention studies involving older clinical populations [57–59]. Our goal in Experiment 2 was to shorten the experimental session time and focus only on executive control performance using an Eriksen Flanker task, thus allowing us to rule out complexity and fatigue as alternate explanations for the null results found in Experiment 1.

### Participants

A total of 220 participants were recruited from undergraduate psychology courses at the University of British Columbia and received course credit for their time. Participants were eligible to take part in the study if they were young adults (under 45 years of age) and physically able to take part in PA. Two participants were excluded due to computer problems that prevented data collection. Our final sample consisted of 218 participants (mean age = 20.11, *SD* = 1.87, 48 male). Participant demographic information can be found in Table 1.

### Procedure

Free-living PA over the past week was assessed using the IPAQ, and executive control was measured using a modified Eriksen Flanker task. Each trial began with a fixation cross in the center of the screen, displayed for 1000ms. Next, flanker arrows (five horizontal arrowheads) were shown for 200ms at the center of the screen, and participants had a maximum of 1500ms (from stimulus onset) to make a response. Participants were asked to use the left and right arrows keys on the keyboard to indicate the pointing direction of the central arrow. Like Experiment 1, arrow directions could either be leftward-congruent (all arrows pointing to the left), leftward-incongruent (center arrow pointing left, flanking arrows pointing right), rightward-congruent (all arrows pointing to the right), or rightward-incongruent (center arrow pointing right, flanking arrows pointing left). All stimulus conditions were equiprobable and presentation order was randomized. Feedback was displayed after each trial with the words “correct”, “incorrect”, or “too slow” depending on the response. At the end of the trial, the fixation cross was shown again for 1500ms before the next trial began. Participants began with 12 practice trials, and the main task consisted of 100 trials. Performance was calculated as the difference in reaction time between incongruent and congruent trials, which was then normalized like in Experiment 1.

### Data analysis

We began by confirming that the congruency manipulation during the flanker task was effective by checking that incongruent trials resulted in longer reaction times than congruent trials using a t-test. The remaining analyses followed the same plan as Experiment 1, where each of the multiple regression models (models 1–3) were used to test the relationship between weekly PA and flanker performance. Regression assumptions were checked by visual inspection of quantile-quantile and normality plots, and no violations were indicated. Furthermore, due to potential issues with collinear predictors, variance inflation factor (VIF) was calculated for all predictors in each of the models. All VIF values were within an acceptable range (< 1.5).

### Results

A paired samples t-test showed that participants took significantly longer to respond to the incongruent trials than the congruent trials, suggesting that the flanker congruency manipulation was effective, *t*(217) = 25.11, *p* < .001. To test our main hypothesis, we tested Models 1–3 using the normalized flanker difference score as the dependent variable. Model 1 showed that none of the predictors were significantly related to flanker performance. Age: *β* = 0.05, 95% CI [-0.10, 0.19], *t*(194) = 0.66, *p* = .51, low-intensity: *β* = 0.02, 95% CI [-0.12, 0.17], *t*(194) = 0.35, *p* = .73, moderate-intensity: *β* = -0.09, 95% CI [-0.23, 0.05], *t*(194) = -1.27, *p* = .21, vigorous-intensity: *β* = 0.04, 95% CI [-0.10, 0.17], *t*(194) = 0.53, *p* = .60. Using Model 2, we tested the relationship between total PA and flanker performance. However, none of the predictors were significantly related to performance. Age: *β* = 0.04, 95% CI [-0.10, 0.19], *t*(196) = 0.56, *p* = .58, total PA: *β* = -0.01, 95% CI [-0.15, 0.13], *t*(196) = -0.14, *p* = .89. Finally, we tested the relationship between ACSM category and executive control using Model 3. Again, none of the predictors were significantly related to flanker performance. Age: *β* = 0.04, 95% CI [-0.10, 0.19], *t*(196) = 0.55, *p* = .58, ACSM: *β* = 0.06, 95% CI [-0.22, 0.34], *t*(196) = 0.44, *p* = .66.

It is possible that PA effects are only observable for the more difficult task conditions, so Model 1 was also tested on congruent and incongruent reaction times separately. However, none of the predictors were significantly related to congruent trial performance. Age: *β* = 0.11, 95% CI [-0.04, 0.26], *t*(194) = 1.43, *p* = .16, low-intensity: *β* = 0.06, 95% CI [-0.08, 0.21], *t*(194) = 0.87, *p* = .39, moderate-intensity: *β* = 0.07, 95% CI [-0.07, 0.21], *t*(194) = 0.96, *p* = .34, vigorous-intensity: *β* = -0.002, 95% CI [-0.14, 0.14], *t*(194) = -0.02, *p* = .98. Additionally, incongruent trial performance also failed to show a relationship with PA. Age: *β* = 0.13, 95% CI [-0.02, 0.28], *t*(194) = 1.73, *p* = .09, low-intensity: *β* = 0.08, 95% CI [-0.06, 0.23], *t*(194) = 1.17, *p* = .25, moderate-intensity: *β* = 0.02, 95% CI [-0.12, 0.16], *t*(194) = 0.26, *p* = .79, vigorous-intensity: *β* = 0.02, 95% CI [-0.12, 0.16], *t*(194) = 0.29, *p* = .77.

### Discussion

Overall, our results failed to support the hypothesis that free-living PA is related to executive control, and the null results found in Experiment 1 were unlikely to be due to task complexity and fatigue effects. It has been suggested that executive control in young adults is highly efficient [60] and it is possible that the benefits of PA do not emerge unless the cognitive task is sufficiently demanding [24,61,62]. This idea is supported by studies examining the impact of aerobic fitness on executive control in pre-adolescent populations, showing that PA benefits are most strongly observed for higher difficulty versions of the flanker task [63,64]. While we are not studying pre-adolescents in the present set of studies, the PA benefit during more difficult tasks may also extend to young adult populations. Our analyses attempted to distinguish high and low difficulty conditions by examining congruent and incongruent trial performance separately, finding no difference between the trial types, however, it is possible that the incongruent condition was still not demanding enough for a PA benefit to be observed.

## Experiment 3

The goal of Experiment 3 to test whether the null results observed in the previous experiments may be due to the task being too easy. The difficulty of the flanker task can be experimentally manipulated by introducing an “incompatible” condition, where the participant is asked to respond in the *opposite* direction of the central arrow [60]. This manipulation has been successfully used to show an association between aerobic fitness and executive control, at least in pre-adolescent populations [63,64].

### Participants

A total of 210 participants were recruited from undergraduate psychology courses at the University of British Columbia and received course credit for their time. Participants were eligible to take part in the study if they were young adults (under 45 years of age) and physically able to take part in PA. Four participants were excluded due to computer problems, declined to report their age, was wheelchair-bound and not physically active, and reported IPAQ activity durations using different (unknown) timescales. Our final sample consisted of 206 participants (mean age = 20.33, *SD* = 2.71, 51 male). Participant demographic information can be found in Table 1.

### Procedure

The experimental setup was identical to Experiment 2 barring the addition of a within-subject compatibility manipulation. During compatible trials, participants were instructed to respond to the pointing direction of the central arrow (same as Experiment 2), and during incompatible trials they were asked to respond in the *opposite* direction of the central arrow using the arrow keys on the keyboard. Compatible and incompatible trials were blocked, and block order was counterbalanced between participants. Each compatibility block contained 100 trials. The congruency manipulation was the same as the previous two experiments, where arrow directions could either be leftward-congruent (all arrows pointing to the left), leftward-incongruent (center arrow pointing left, flanking arrows pointing right), rightward-congruent (all arrows pointing to the right), or rightward-incongruent (center arrow pointing right, flanking arrows pointing left).

### Data analysis

We began by confirming that the congruency and compatibility manipulations were effective using a 2-way ANOVA. The remaining analyses followed the same plan as the previous experiments, where the multiple regression models (models 1–3) were used to test the relationship between weekly PA and flanker performance for each of the compatibility conditions. Regression assumptions were checked by visual inspection of quantile-quantile and normality plots, and no violations were indicated. Furthermore, due to potential issues with collinear predictors, variance inflation factor (VIF) was calculated for all predictors in each of the models. All VIF values were within an acceptable range (< 1.5).

### Results

**Fig 1:**
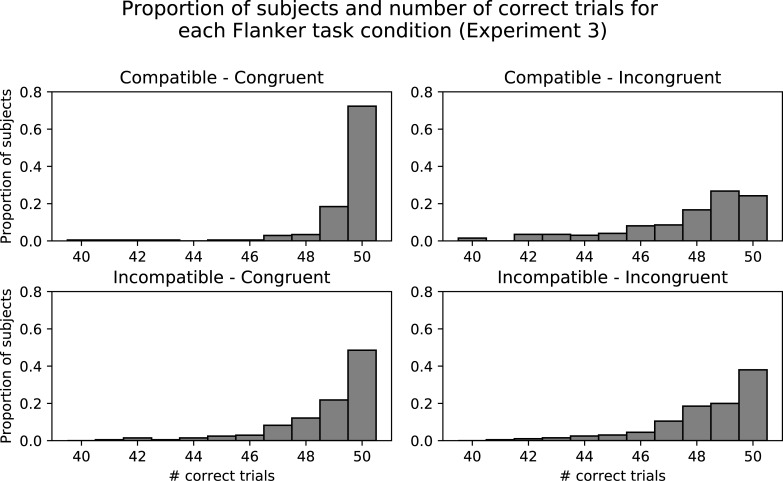
Histograms of task accuracy in each of the experimental conditions during Experiment 3. A large proportion of participants achieved perfect accuracy (50 correct trials out of 50), with most performing close to ceiling, suggesting the Flanker task may not have been difficult enough.

As a manipulation check, reaction time was submitted to a 2 (compatibility: compatible, incompatible) x 2 (congruency: congruent, incongruent) within-subjects ANOVA to ensure that 1) incongruent trials were more difficult than congruent trials, and 2) that incompatible trials were more difficult than compatible trials. The reaction time means (and standard deviations) for the compatible-congruent condition: 470.67ms (65.36), compatible-incongruent: 520.86ms (68.01), incompatible-congruent: 506.29ms (75), incompatible-incongruent: 531.52ms (87.89). A significant interaction between compatibility and congruency was found, *F*(1, 205) = 88.44, *p* < .001, η_p_^2^ = 0.30. There was a significant main effect of compatibility, *F*(1, 205) = 32.71, *p* < .001, η_p_^2^ = 0.14, as well as a significant main effect of congruency, *F*(1, 205) = 469.94, *p* < .001, η_p_^2^ = 0.70. The observed data pattern is as predicted, with congruent trials faster than their incongruent counterparts, and compatible trials faster than incompatible trials, suggesting a successful difficulty manipulation. However, simple main effects analysis of the interaction, with Bonferroni adjustment for multiple comparisons, showed a significant difference between compatible and incompatible conditions for the congruent trials, *t*(248.63) = 8.37, *p* < .001, but not for the incongruent trials, *t*(248.63) = 2.50, *p* = .08. This is likely due to a ceiling effect in task performance, rather than the difficulty manipulation only working for congruent trials, and is supported by looking at histograms of task accuracy for each condition (Fig 1). The graphs show that the vast majority of participants achieved near perfect accuracy in all conditions. Perfect performance can place an upper bound on reaction time. In other words, due to the ease of the overall Flanker task, participants needed no more than 531.52ms (on average) to complete even the most difficult trials (incompatible-incongruent), and this cap on reaction time likely prevented a significant simple main effect from being observed in the incongruent trials. The overall data pattern suggests that the difficulty manipulation was effective, but the Flanker task itself may not be difficult enough, thus causing a ceiling effect that masks the true difficulty of the incompatible condition.

For the compatible condition, Models 1–3 were tested using the normalized flanker difference score as the dependent variable, which should replicate our findings from Experiment 2. Model 1 showed that none of the predictors were significantly related to compatible flanker performance. Age: *β* = -0.03, 95% CI [-0.17, 0.12], *t*(190) = -0.37, *p* = .71, low-intensity: *β* = 0.03, 95% CI [-0.12, 0.18], *t*(190) = 0.45, *p* = .65, moderate-intensity: *β* = 0.01, 95% CI [-0.16, 0.17], *t*(190) = 0.08, *p* = .94, vigorous-intensity: *β* = 0.001, 95% CI [-0.16, 0.16], *t*(190) = 0.01, *p* = .99. Using Model 2, we tested the relationship between total PA and compatible flanker performance. However, none of the predictors were significantly related to performance. Age: *β* = -0.03, 95% CI [-0.17, 0.12], *t*(192) = -0.37, *p* = .71, total PA: *β* = 0.02, 95% CI [-0.12, 0.17], *t*(192) = 0.34, *p* = .74. Finally, we tested the relationship between ACSM category and executive control using Model 3. Again, none of the predictors were significantly related to compatible flanker performance. Age: *β* = -0.03, 95% CI [-0.17, 0.11], *t*(192) = -0.39, *p* = .70, ACSM: *β* = 0.02, 95% CI [-0.28, 0.33], *t*(192) = 0.16, *p* = .88.

For the incompatible condition, Models 1–3 were tested using the normalized flanker difference score as the dependent variable. Model 1 showed that none of the predictors were significantly related to incompatible flanker performance. Age: *β* = 0.09, 95% CI [-0.06, 0.23], *t*(190) = 1.20, *p* = .23, low-intensity: *β* = -0.01, 95% CI [-0.16, 0.14], *t*(190) = -0.14, *p* = .89, moderate-intensity: *β* = -0.05, 95% CI [-0.21, 0.12], *t*(190) = -0.57, *p* = .57, vigorous-intensity: *β* = 0.08, 95% CI [-0.07, 0.24], *t*(190) = 1.05, *p* = .30. Using Model 2, we tested the relationship between total PA and incompatible flanker performance. However, none of the predictors were significantly related to performance. Age: *β* = 0.08, 95% CI [-0.06, 0.22], *t*(192) = 1.10, *p* = .27, total PA: *β* = 0.03, 95% CI [-0.12, 0.17], *t*(192) = 0.36, *p* = .72. Finally, we tested the relationship between ACSM category and executive control using Model 3. Again, none of the predictors were significantly related to incompatible flanker performance. Age: *β* = 0.08, 95% CI [-0.06, 0.22], *t*(192) = 1.10, *p* = .26, ACSM: *β* = 0.07, 95% CI [-0.23, 0.37], *t*(192) = 0.48, *p* = .63.

### Discussion

Our results failed to show a relationship between free-living PA and executive control, even after introducing a more difficult task condition. Furthermore, no difference was observed when looking at separate PA intensity levels versus total PA, nor was there a difference between different IPAQ scoring methods. One potential issue is the use of the Flanker task. The reaction time data pattern suggests that the difficulty manipulation was effective, with incompatible trials resulting in slower overall responses than compatible trials. However, task accuracy suggests a ceiling effect, capping both accuracy and reaction time during the task. That is to say that while a more difficult task condition was introduced, the Flanker task itself may still not be difficult enough for PA effects to be observed. Furthermore, overall cognitive function and processing speed are generally better in young adults than other age groups [52], and some studies have shown that PA benefits executive control only in older adults [19,65]. The ceiling effect may be due to studying a young adult age group where executive control functions well enough that either no benefit arises from increased PA, or an extremely demanding cognitive task is required for the effects to be observed.

## General discussion

Overall, our results show that self-reported PA over the past week, at any intensity level, does not appear to have a substantive impact on executive control. Our control studies (Experiments 2 and 3) show that the null findings were not due to fatigue and task complexity, or low cognitive demands of the task. While it is certainly possible that there is no relationship between free-living PA and executive control in younger adults, there are several important factors that need to be considered with regard to our null findings.

Studies of long-term chronic PA have shown improvements to executive control [8,66] and similarly very short-term acute PA has also been shown to be beneficial [2,3]. Those studies exist at opposite ends of the temporal continuum and it is possible that mid-term PA, such as the type studied here, may be too recent to see the chronic benefits from improved aerobic fitness and cardiovascular health, and at the same time too distal to see improvements due to acute physiological arousal. However, we did not control for the effects of longer-term habitual PA or aerobic fitness, which may have a larger impact on cognition than PA at short timescales [67,68]. Other research in our lab has demonstrated that increased levels of free-living PA over the past two days is associated with improved performance on an academic exam, suggesting that PA benefits may be observable only when looking at PA that occurred very close to the time of cognitive assessment, making it more similar to acutely measured activity. In other words, just like there is an inverted-U relationship between PA intensity and cognition [24,25,27,28], a similar relationship may exist with PA recency, with effects only observable for very recent or very long-term activity.

There may be factors that mediate the relationship between free-living PA and executive control. A recent study showed that frequency of PA is related to improved executive control, but that this relationship is mediated by efficiency of cerebral blood-flow regulation [20]. Aerobic fitness may be a better predictor of executive control than measures of moderate-to-vigorous physical activity [68], which may explain why chronic PA studies have found associations with executive control given that increased levels of habitual long-term activity would likely increase overall aerobic fitness. Sleep efficiency has also been shown to mediate the relationship between objectively measured PA and executive control [69]. Finally, some research has shown that self-reported PA is related to executive control only in lean, but not obese, individuals [36]. While these studies have assessed different timescales, it would be worth investigating the mediating effects of health-related metrics such as sleep, fitness, and obesity.

We used an effect size estimate of 0.20 when conducting the power analysis to determine required sample size for the studies. There is little research studying the impact of self-reported free-living PA on executive control in young adults, however, a review of the literature, using different PA and cognitive measures, generally shows larger effect sizes than the conservative 0.20 that we used for the power analysis. For example, studies have found that the association between PA and executive control ranges from 0.22 to 0.41 [20,29,36,69], and moderate-intensity PA and attention capacity to be 0.30 [15]. Meta-analyses show the relationship between PA interventions and academic achievement in high school aged adults to be 0.24 [70], acute PA and cognitive function to be 0.20 [1], and fitness and cognitive function in college-aged adults to be 0.64 [37]. Furthermore, Experiments 2 and 3 had a large majority of female participants, and some studies suggest that females show larger effect sizes than males [67,71]. Granted that some studies have shown smaller relationships between PA and executive control, with effect sizes around 0.10 [30,68], we remained conservative in our estimated effect size relative to the majority of the PA literature to avoid being underpowered. However, our observed effect sizes for executive control were as low as 0.001 in some models, suggesting that we may be underpowered for detecting such small effects. It should be noted that many of the confidence intervals around our point estimates were wide enough to contain our estimated effect of 0.20, leaving open the possibility of an effect of that size in the population. If, however, the population effect sizes are actually as small as observed, this raises a question of practical significance, even if statistical significance was not found. For example, our models for the incompatible flanker task in Experiment 3 showed effect sizes of 0.01, 0.05, and 0.08 on a standardized metric for low, moderate and vigorous intensity PA. In the raw score metric, each unit increase in MET-minutes/week would correspond to reaction differences (between congruent and incongruent trials) of 4.56 × 10^−5^, 1.15 × 10^−3^, and 8.77 × 10^−4^ milliseconds, which are too small to be of any meaningful importance.

Various PA measurement-related issues may contribute to the differences between our null findings and the significant results reported in the PA literature. A number of studies have used the IPAQ, but only reported PA duration, rather than duration weighted by intensity [16–18,20,21]. Not only does this fail to account for the importance of PA intensity, but it may also alter the psychometric properties of the measurement instrument. The IPAQ was originally designed and validated as a measure of MET-minutes per week [40], and it is unknown how reliability and validity are affected when only the time-based PA metrics are utilized. We did not observe significant relationships when using the ACSM time-based coding method for the IPAQ, however, this may be due to differences in cognitive task used, timescale differences, or not considering potential mediators as previously discussed. Furthermore, some IPAQ studies have only used the short-form of the questionnaire [20,30,36,43], which has been shown to have relatively weak psychometric properties, with low correlations against objective measures and a tendency to overestimate activity levels [45]. Overall, there is no single standard for collecting and handling self-reported PA data, and this can lead to differences in the accuracy, reliability, and validity of the measures used, resulting in some studies reporting significant associations, and others demonstrating null relationships.

Finally, research has shown that self-reported PA may be inaccurate, have low reliability and low validity compared to more direct measures of PA [72–77], while objective PA measures show stronger correlations with anthropometric variables, such as BMI, than self-report questionnaires [78]. Therefore, inaccuracies stemming from subjective reporting may explain our lack of significant findings. The choice between subjective and hardware-based PA recording is not straightforward, however, as objective methods have environmental limitations (for example, they cannot measure water-based activities, or activities where the body remains stationary such as cycling), and require high wear-time to be accurate [79]. Furthermore, many studies have found significant associations between self-reported PA and cognitive function [8,17,18,20,22,36,80,81], suggesting our null results are not solely due to the use of subjective reporting. One possibility is that our method of cognitive assessment (behavioral performance on the ANT and Flanker tasks) may not be sensitive enough to show significant relationships with free-living PA. Across a number of PA time windows (acute, chronic, free-living), many studies have shown no relationship between PA and behavioral measures of cognitive performance, whereas a significant relationship is seen when using neuroimaging techniques to assess cognition [23,32,82–85]. This difference between behavioral and neuroimaging results suggests that a more sensitive measure of cognitive performance may be needed to observe PA benefits in young adult populations. Additionally, the Flanker task is a commonly used measure of executive control and other studies have demonstrated improved performance due to increased levels of PA [32,86,87]. While these significant findings differ from the null results presented here, the discrepancy can again be explained methodologically, as those studies found neuroelectric changes during the Flanker task, while our focus was on behavioral Flanker performance.

Overall, there is a paucity of research on the effects of self-reported free-living PA, and it is difficult to make direct comparison between the present study and much of the PA literature. This is, by and large, due to published studies not making a clear distinction between chronic and free-living timescales [19,22,88,89], and even combining multiple timescales into a single equally-weighted composite measure [65]. Moving forward, we suggest that the distinction is vital for understanding the impact of PA on executive control, as the timescale of assessment can have a markedly different impact on strength and direction of association.

## Supporting information

S1 Dataset IPAQ and computer-task data for all experiments. (ZIP)
